# On the Influence of the Mineral Kingdom on Disease

**Published:** 1853-07

**Authors:** J. P. Hiester

**Affiliations:** Reading, Pa.


### MEDICAL EXAMINER,

AND

### RECORD OF MEDICAL SCIENCE.

NEW SERIES.—NO. CIII.—JULY, 1853.

### ORIGINAL COMMUNICATIONS.

### *On the Influence of the Mineral Kingdom on Disease.*

By J. P. Hiester, M. D.

Reading, Pa., June 8th, 1853.

To the Editors of the Medical Examiner:

In the January No. for 1852, you did me the honor to publish a short communication on the above subject, which had been read before the Berks County Medical Society. I there endeavored to show, by established facts and a course of reasoning, the influence exerted by inorganic matter, as presented in the natural state, upon organized beings.

A sufficient number of facts were adduced to prove the influence of soil upon vegetables, and the probable effect of soil and water, or *minerals in solution*, upon the lower orders of animals and fishes. From analogical reasoning it was conjectured, that the higher orders of animals were also subjected to a greater influence by mineral agents than we are, in the present state of our knowledge, prepared to admit.

It was farther concluded, from the activity of many of the mineral agents that are known to enter into the composition of the animal organism, either necessarily or adventitiously, that their influence might, and probably did, produce decidedly pathological conditions, or at least such changes as would strongly predispose to disease ; and that even deficiencies of many mineral substances in certain soils, might produce such conditions or predispositions. Iodine, which late observation has shown to be far more extensively distributed than was formerly supposed, was particularly referred to as one of these active agents.

In verification of the above conjectures, I would beg leave to call the attention of your readers to the following well-authenticated and interesting facts contained in a communication made to the French Academy of Sciences, extracted from the May No., 1853, of the Journal de Médecine et de Chirurgie Pratique.

"M. Chatin communicated to the Academy the following extremely curious facts :

"Fully and Saillon are two contiguous villages situated in the midst of the vineyards that border the right bank of the Rhone. Fully, where the whole population is goiterous, is remarkable for its great number of Cretins ; Saillon, on the contrary, was renowned in the Vallais for the fine health of its inhabitants, who were scarcely ever attacked by goitre, and more rarely still by cretinism. The contrast was the more marked as the conditions of altitude, aëration, exposure, &c., are as nearly alike as is possible in the two villages.

"But within a few years Saillon has lost the happy exemption which it enjoyed ;—goitre and cretinism have increased to a degree that scarcely leaves Fully any room for envy. The observations made by M. Moulin, President of Saillon, prove that the progress of goitre and cretinism date from the time when, in defiance of the counsel of M. Barmont, brother of the Swiss Ambassador at Paris, the commune took the water, destined for the use of the village, formerly received from a lower point of the torrent, (the Salente,) from a point where it is precipitated in a cascade from the glacier of the mountain. Between these two points, a strong thermal spring (temperature about 28 C.) falls into the torrent, constituting nearly a sixtieth part of its waters.

"From my analysis it results :

"1. That water taken from above the point where the hot spring enters the torrent, and which is that at present used in Saillon, is destitute of *Iodine*, like that of Fully, and of the greater part of the districts of the Vallais.

"2. That the water taken from the point whence Saillon was formerly supplied is more iodized than the water used in Paris.

"3. That the water of the thermal spring above referred to, is a true mineral water, containing at least sixty times more iodine than the water of Paris, and that of most of the countries where goitre is unknown.

"These facts go to prove :

"1. The existence and nature of a *local* cause of goitre and cretin'sm.

"2. The possibility of introducing iodized water as a prophylact'c in these maladies, both as a regimen for man, and for animals p:oducing meat, milk, &c."

